# Low Frequency Electrical Stimulation Attenuated The Epileptiform Activity-Induced Changes in Action Potential Features in Hippocampal CA1 Pyramidal Neurons

**DOI:** 10.22074/cellj.2018.5443

**Published:** 2018-05-28

**Authors:** Zahra Ghasemi, Nima Naderi, Amir Shojaei, Nooshin Ahmadirad, Mohammad Reza Raoufy, Javad Mirnajafi-Zadeh

**Affiliations:** 1 Department of Physiology, Faculty of Medical Sciences, Tarbiat Modares University, Tehran, Iran; 2 Department of Pharmacology and Toxicology, School of Pharmacy, Shahid Beheshti University of Medical Sciences, Tehran, Iran

**Keywords:** Action Potential, Brain Stimulation, Hippocampus, Rat

## Abstract

**Objective:**

Electrical low frequency stimulation (LFS) is a new therapeutic method that moderates hyperexcitability during epileptic states. Seizure occurrence is accompanied by some changes in action potential (AP) features. In this study, we investigated the inhibitory action of LFS on epileptiform activity (EA) induced-changes in AP features in hippocampal CA1 pyramidal neurons.

**Materials and Methods:**

In this experimental study, we induced EA in hippocampal slices by increasing the extracellular potassium (K^+^) concentration to 12 mM. LFS (1 Hz) was applied to the Schaffer collaterals at different pulse numbers (600 and 900) at the beginning of the EA. Changes in AP features recorded by whole-cell patch clamp recording were compared using phase plot analysis.

**Results:**

Induction of EA depolarized membrane potential, decreased peak amplitude, as well as the maximum rise and decay slopes of APs. Administration of 1 Hz LFS at the beginning of EA prevented the above mentioned changes in AP features. This suppressive effect of LFS depended on the LFS pulse number, such that application of 900 pulses of LFS had a stronger recovery effect on AP features that changed during EA compared to 600 pulses of LFS. The constructed phase plots of APs revealed that LFS at 900 pulses significantly decreased the changes in resting membrane potential (RMP), peak amplitude, and maximum rise and decay slopes that appeared during EA.

**Conclusion:**

Increasing the numbers of LFS pulses can magnify its inhibitory effects on EA-induced changes in AP features.

## Introduction

Electrical low frequency stimulation (LFS), an important pattern of deep brain stimulations, has been evaluated as a potential therapeutic strategy to moderate hyperexcitability that appears during neurological disorders such as epilepsy (1-4). High frequency stimulation is used in a therapeutic manner; however, LFS has a lower risk of damage to brain structures, relative safety, and reversibility (1, 3). This method, introduced as a new manner to treat uncontrolled epilepsy, is used in both experimental animal models (3-6) and epileptic patients (7-9). The inhibitory action of LFS on kindled seizures (5, 10) as well as epileptiform activity (EA) in brain slices (11) has been shown in previous studies.

Seizure occurrence is accompanied by changes in neural excitability. Different factors, including the features of action potential (AP), can be used as an index of neural excitability. APs, as the main factor for transferring information in the nervous system, are crucial components that regulate brain activity (12). Therefore, the exact timing and features of APs are decisive for dynamic encoding and processing of information in the neuronal network (13, 14). 

Previous studies on kindled animals (15, 16) and in vitro isolated rodent brains (17) showed that seizures could induce alterations in AP features. Research showed that the augmented activity during seizures was accompanied by decrements in amplitude, depolarizing and repolarizing slopes, and increments in frequency and half-width of the APs. In addition, the resting membrane potential (RMP) and threshold of APs shifted to more depolarized values in the epileptic states (15, 17). 

It would be beneficial to assess the effect of LPS on AP features generated during epilepsy considering its potential for treating epilepsy. In order to explore the effect of LFS on AP features and considering the important role of hippocampus in the epilepsy (18), EA was induced in the hippocampal brain slices via increasing extracellular potassium (K^+^) concentrations. The high-K^+^ model is a proper model for induction of EA in brain slices because in vivo studies show elevated levels of [K^+^]o to 12 mM in the hippocampus during the seizure state. Any impairment in glial cells which leads to increments in [K^+^]o can induce seizures. In addition, it has been shown that increasing extracellular K^+^ concentrations, as a well-known model in epilepsy researches, results in prolonged neuronal depolarization and large ionic changes in hippocampal compartments (19-22). In this study, we try to determine the effect of 1 Hz LFS applied to the Schaffer collaterals on the EA-induced changes in AP features. 

## Materials and Methods

### Experimental animals

In this experimental study, we housed 4-6 week old male Wistar rats under standard laboratory conditions of 22-25°C, 12 hour light/dark cycle, and free access to food and water. All experimental procedures and the care of animals were in accordance with the guidelines of the Institutional Animal Ethics Committee at the Faculty of Medical Sciences, Tarbiat Modares University, and were completely in line with the NIH Guide for the Care and Use of Laboratory Animals.

### Preparation of brain slices and whole-cell recording 

In this experiment, whole cell current clamp recordings were performed on the hippocampal CA1 pyramidal neurons of rats. The rat brains were quickly removed and placed in a cold cutting artificial cerebrospinal solution (ACSF) that contained (in mM): 238 sucrose, 2.5 KCl, 0.5 CaCl_2_, 2 MgSO_4_, 1 NaH_2_PO_4_, 26.2 NaHCO_3_ and 11 D-glucose adjusted to a pH=7.3-7.4 with 95% oxygen, 5% carbon dioxide, and an osmolarity to 290-300 mOsm. Horizontal slices (400 µm) of the right hippocampus that contained the entorhinal cortex due to its involvement in seizure generation (23) were prepared using a vibratome (VT1200S, Leica, Nussloch, Germany). The prepared slices were transferred to a Gibbs chamber that contained 32-35oC standard ACSF (sACSF) composed of (in mM): 125 NaCl, 3 KCl, 2 CaCl_2_, 2 MgSO_4_, 1.25 NaH_2_PO_4_, 25 NaHCO3, and 10 D-glucose at a pH of 7.3-7.4 and osmolarity of 290-300 mOsm for 1 hour. Then, prior to transfer to a submerged recording chamber, we maintained the slices at room temperature for at least 30 minutes. The submerged recording chamber was continuously superfused with sACSF at a rate of 2 ml/minutes. This chamber was located on the stage of an upright microscope (Axioskop 2 FS MOT, Carl Zeiss, Germany) equipped with an infrared CCD camera (IR-1000, MTI, USA) to visualize hippocampal CA1 pyramidal neurons. 

Intracellular activity was recorded from the CA1 pyramidal cells using borosilicate glass pipettes (1.5 mm O.D. and 0.86 mm I.D., Sutter, USA) filled with an intracellular solution that contained the following (in mM): 120 K-gluconate, 6 NaCl, 6 CaCl_2_, 2 MgCl_2_, 2 Mg ATP, 0.5 Na GTP, 12 phosphocreatine, 5 EGTA, and 10 HEPES with an equilibrated pH=7.3-7.4 and osmolarity of 290-300 mOsm.

The tip resistance of the micropipettes pulled via a microelectrode puller (P-97, Sutter Instrument, USA) was 4-6 MΩ. The electrode capacitance and series resistance were compensated. The cells that had changes in series resistance of more than 25% were rejected from data collection. Signals were acquired using a multiclamp 700B amplifier and digitized with a Digidata 1440 A/D converter (Molecular Devices, CA, USA). All recordings were acquired at 10 kHz and digitized at 2 kHz. Data were saved in a PC and analyzed offline using pCLAMP 10 software (Molecular Devices, CA, USA). 

**Fig.1:**
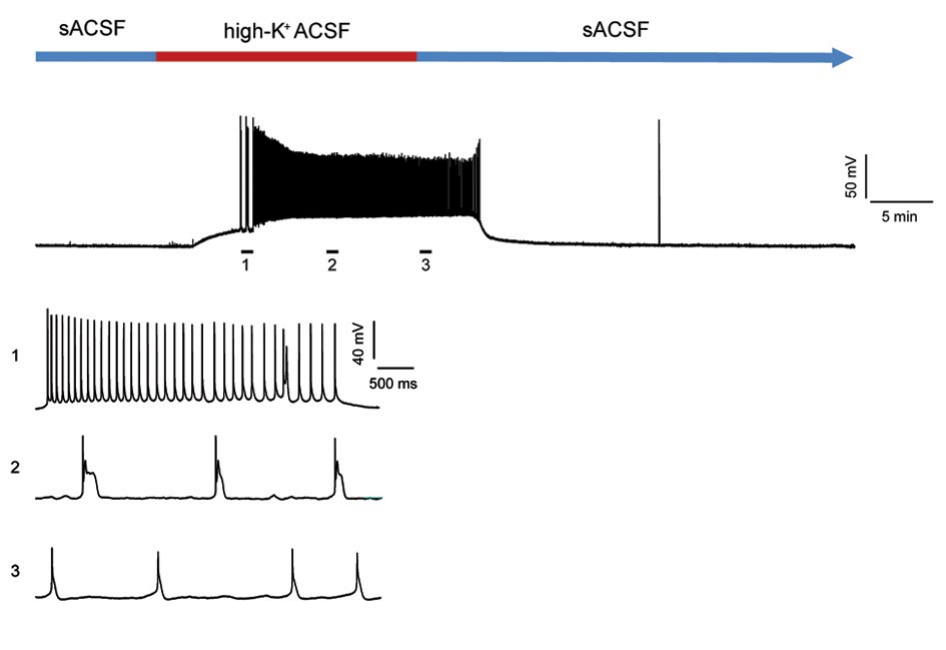
Representative whole cell patch clamp recordings of spontaneous firing of a CA1 pyramidal neuron induced by a 20 minutes perfusion of high potassium artificial cerebrospinal solution (high-K^+^ ACSF). Numbers indicate the time points in which the sample voltage traces are shown at an expanded time scale.

EA was induced by applying a high potassium ACSF (high-K^+^ ACSF) that contained (in mM): 116 NaCl, 12 KCl, 1 NaH_2_PO_4_, 25 NaHCO_3_, 10 D-glucose, 2 CaCl_2_, 2 MgSO4 with a pH=7.3-7.4 and osmolarity to 290-300 mOsm. At first, we recorded the intracellular activity of the cells in the presence of sACSF for 10 minutes, after which the slices were exposed to high-K^+^ ACSF for 20 minutes. Spontaneous firing of hippocampal CA1 pyramidal neurons following application of high-K^+^ ACSF significantly increased while these neurons had no or very low spontaneous firing in sACSF. Spontaneous firing in the range of 0.2 Hz to 100 Hz that appeared 5.64 ± 0.42 minutes after superfusion of high-K^+^ ACSF were persistent for at least 20 minutes after high-K^+^ ACSF washout from the recording chamber (Fig .1).

LFS was applied to the Schaffer collaterals as 600 (LFS600) and/or 900 (LFS900) square wave pulses of 0.1 mseconds at a frequency of 1 Hz. LFS was delivered at the beginning of the EA at an intensity of 1.5 times more than the intensity sufficient to elicit an evoked EPSP of at least 5 mV amplitude.

We compared four experimental groups in this study. The high-K^+^ ACSF (HK) group had slices that were superfused by high-K^+^ACSF and no electrical stimulation. The LFS600 group received 600 pulses of LFS delivered to the Schaffer collaterals at the beginning of the EA. In the LFS900 group, the slices received 900 pulses of LFS at the beginning of the EA. In the LFS groups, LFS (either 600 or 900 pulses) was applied to the slices without high-K^+^ ASCF. 

**Fig.2:**
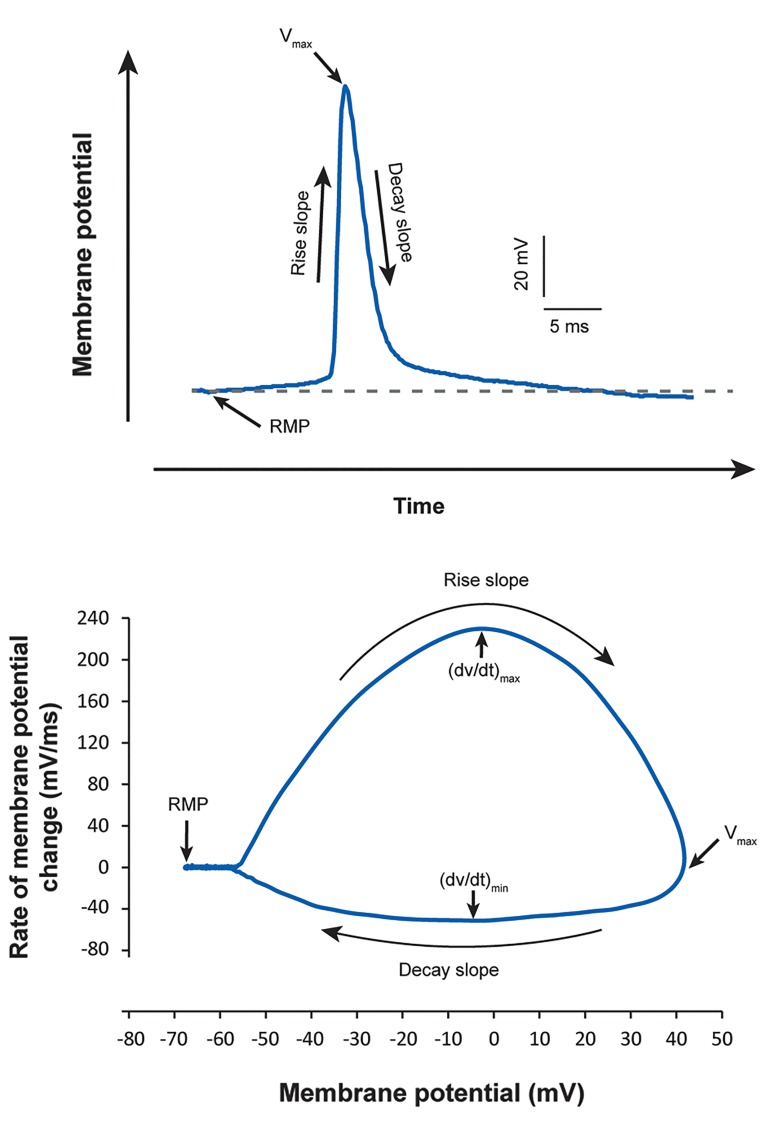
Phase plot analysis of action potentials (APs). Top: AP recorded from a CA1 pyramidal neuron. Bottom: Phase plot that shows different parts of the AP presented at top.

Phase plots were constructed to characterize and compare the effect of the increased extracellular K^+^ concentration on the features of AP recorded from hippocampal CA1 pyramidal neurons. In phase plot analysis, the time derivative of the membrane potential (dV/dt) was plotted versus the membrane potential (Fig .2).

In all experimental groups, we evaluated changes in RMP, peak amplitude of the APs, and maximum rise and decay slopes of the APs for 5 minutes in 3 phases: immediately after EA initiation, at the middle of EA, and after high-K^+^ ACSF washout with sACSF. We did not observe any AP after 5 minutes of washout in most of the recorded slices. Therefore, we considered the first 5 minutes immediately after the washout for measuring and evaluating the changes in AP (Fig .1). 

### Statistical analysis

All obtained results were analyzed via pCLAMP(version 10, Molecular Devices, CA, USA) and MATLAB (MathWorks) software. Results are presented as means ± SEM. Different experimental groups were statistically compared using one-way or two-way repeated measures ANOVA followed by Tukey’s post-hoc test using GraphPad Prism software (version 6.01, CA, USA). P<0.05 was considered statistically significant. In each experiment, n represented the numbers of cells and/or slices prepared from at least 3 rats. 

## Results

**Fig.3:**
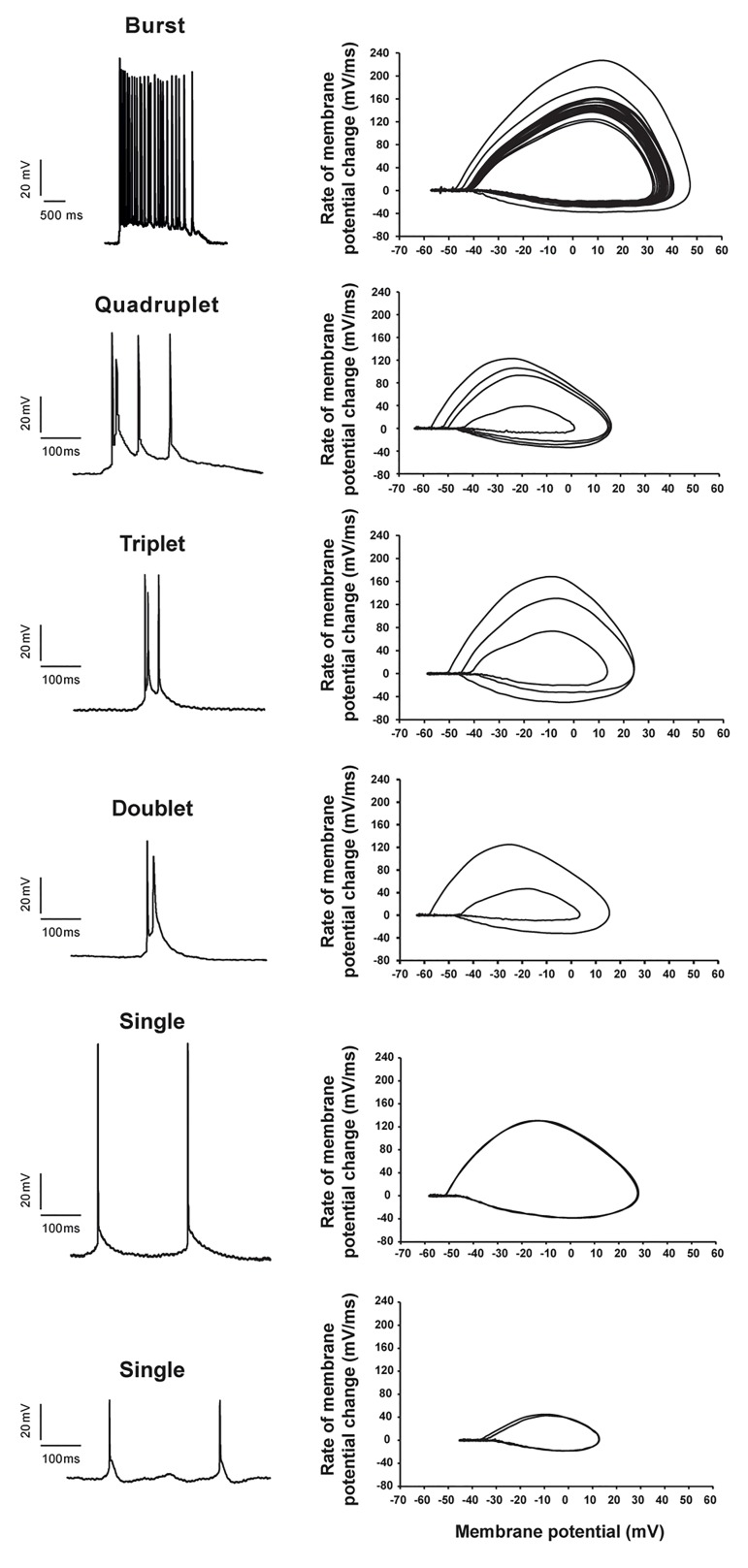
Different patterns of action potentials (APs) of hippocampal CA1 pyramidal neurons that appeared during epileptiform activity (EA) by high potassium artificial cerebrospinal solution (high-K^+^ ACSF). Left: Sample recordings of various AP patterns. Right: Relative phase plots of each voltage sample that illustrates the changes in AP features during EA.

We observed that approximately 5 minutes after administration of high-K^+^ ACSF the CA1 pyramidal cells spontaneously fired the APs. Following a paroxysmal depolarization shift, EA initiated with several bursts of APs followed by the appearance of uneven APs of various amplitudes and multiple spikes that included doublets, triplets, and quadruplets (Fig .1). The AP parameters in the second and third spikes of the doublets and triplets changed such that there was a significant reduction in Vmax, and the rise and decay slopes (Fig .3).

**Fig.4:**
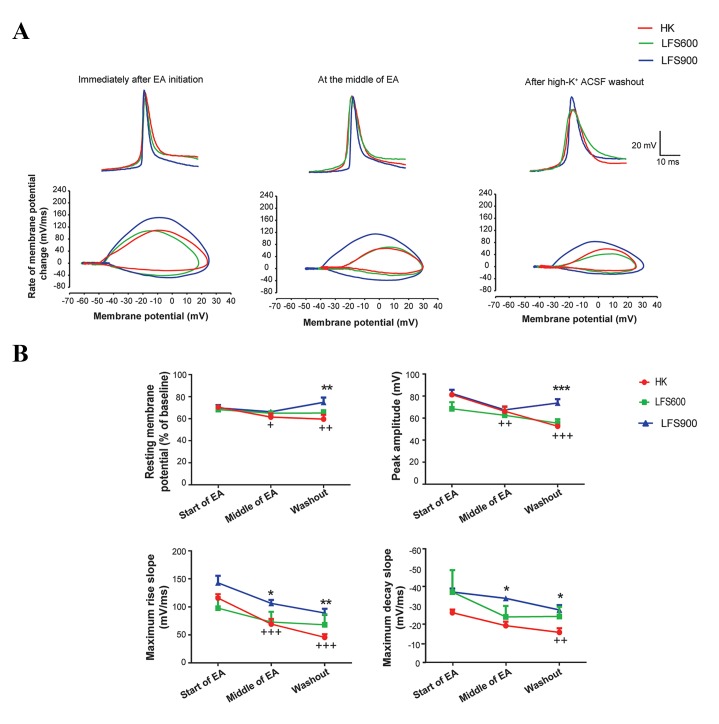
Effect of different pulse numbers of 1 Hz low frequency stimulation (LFS) applied at the beginning of epileptiform activity (EA) on action potentials (APs). A. Phase plot analysis that illustrates the effect of LFS on AP parameters evaluated immediately after initiation of EA, during the middle of EA, and after high potassium artificial cerebrospinal solution (high-K^+^ ACSF) washout and B. Effect of LFS on APs, including resting membrane potential peak amplitude, maximum rise slope, and maximum decay slope measured at three time points during EA.

CA1 pyramidal cells had a mean RMP of -69.3 ± 1.79 mV. Increasing the extracellular potassium concentration ([K]_o_=12 mM) significantly depolarized the RMP to -56.8 ± 1.78 mV at the beginning of the EA and changed it to the most depolarized value of -43.2 ± 2.75 mV during EA (one-way ANOVA, F_(2.27)_=36.84, P<0.001, n=8). Changes in (2,27) RMP were normalized according to the baseline. Figure 4 shows that the application of 900 pulses of LFS significantly prevented HK induced depolarization of RMP after high-K^+^ ACSF washout. This parameter significantly increased from 60% in the HK group to 75% of baseline in the LFS group when measured after the washout (P<0.01, n=7). 

Two-way repeated measure ANOVA demonstrated a significant interaction of different time points of measurements and experimental groups for RMP (F_(4.34)_=3.57, P<0.05). (4,34) Although there was a significant difference in RMP among the different time points (F_(2.34)_=3.8, P<0.05), this parameter (2,34) showed no significant difference between the experimental groups (F_(2.17)_=1.87, P=0.18).

The maximum rise slope of the APs at the beginning of EA was 115.7 ± 7.01 mV/mseconds. This parameter significantly decreased to 69.24 ± 9.21 mV/mseconds when measured in the middle of the EA (P<0.001, n=8) and to 45.52 ± 5.72 mV/mseconds after high-K^+^ ACSF washout (P<0.001, n=8) in the HK group (Fig .4). 

Two-way ANOVA showed no significant interaction of the different time points and experimental groups. However, there was a significant difference for maximum rise slope between the HK and LFSs groups (F_(2.18)_=5.1, P<0.05) as well as different time points of measurements (F_(2.36)_=35.88, P<0.001). Administration of 900 pulses of LFS at the beginning of EA significantly increased maximum rise slope during EA when this parameter was measured at the middle of EA (from 69.24 ± 9.21 mV/ mseconds to 106.2 ± 5.97 mV/mseconds, P<0.05, n=7) and when this parameter was measured after high-K^+^ ACSF washout (from 45.52 ± 5.72 mV/mseconds to 89.03 ± 7.62 mV/mseconds, P<0.01, n=7) (Fig .4). 

The APs of CA1 pyramidal neurons generated after the application of high-K^+^ ACSF had a mean peak amplitude of 81.26 ± 2.43 mV at the beginning of EA. This parameter significantly attenuated to 66.24 ± 4.36 mV when measured at the middle of EA (P<0.01, n=8) and 52.66 ± 2.11 mV after high-K^+^ ACSF washout (P<0.001, n=8, Fig .4). Two-way repeated measures ANOVA revealed significant interactions between different experimental groups and time points for the peak amplitude of APs (F_(4.36)_=4.24, P<0.01). This parameter also showed significant differences between time points (F_(2.36)_=20.75, P<0.001) and experimental groups (F_(2.18)_=5.58, P<0.05). Application of 600 pulses of LFS had no significant effect on HK induced changes in peak amplitude. However, 900 pulses of LFS significantly increased peak amplitude of APs to 73.73 ± 3.46 mV when measured after high-K^+^ ACSF washout (P<0.001, n=7, Fig .4). 

The maximum decay slope value of the APs that appeared immediately after EA initiation was -26.77 ± 1.53 mV/mseconds. This parameter decreased during EA to -16.3 ± 2.17 mV/mseconds when assessed after high-K^+^ ACSF washout (P<0.01, n=8, Fig .4). Although there was no significant interaction of different time points and experimental groups (F_(4.36)_=0.99, P=0.42), this parameter showed significant differences between various time points (F_(2.36)_=17.15, P<0.001) and experimental groups (2,36) (F=4.49, P<0.05).

Application of LFS at 900 pulses significantly augmented the maximum decay slope of APs to -34.39 ± 1.26 mV at the middle of EA and to -28.2 ± 2.5 after high-K^+^ washout (P<0.05, n=7, Fig .4).

Application of LFS alone did not have any significant effect on the measured electrophysiological parameters. 

## Discussion

In the present study we described the features of AP in CA1 pyramidal neurons during 3 phases of high-K^+^ ACSF induced EA. Phase plot analysis revealed that application of LFS at the beginning of EA reduced the changes in AP features that appeared during EA. 

Our results agreed with other studies where LFS applied at 1 Hz had an inhibitory effect on EA in acute brain slices (11, 24, 25). Our data also showed that higher numbers of LFS pulses (900 vs. 600) had a stronger suppressive effect on the AP properties generated during EA. These results agreed with previous studies where consecutive application of 1 Hz LFS for 15 minutes was necessary to inhibit seizures in kindled rats (10) and in exerting its antiepileptogenic effect (26). 

An association existed between administration of high-K^+^ ACSF and the generation of APs with lower peak amplitude, and slower rise and decay slopes. Voltage- gated Na+ and K^+^ channels are the main determinants of AP generation and neuronal excitability in the central nervous system (27-30). An abnormality in their functions causes hyperexcitability and occurrence of seizure activity (20, 31-34). Na+ channels are responsible for the rising phase of APs. The decay phase of APs is mediated through fast activating A-type K^+^ channels as well as delayed rectifier K^+^ channels (13, 35). Elevation of extracellular K^+^ concentrations decrease the driving force of outward K^+^ currents behind AP repolarization (17). Subsequently, the slower decay slope and longer duration of APs is observed following reduction of outward K^+^ currents. Reduction of the outward K^+^ currents results in a depolarized RMP trough that affects the currents which regulate RMP. Our results have indicated that EA led to progressive membrane depolarization. Depolarized RMP can inactivate fast Na+ channels that are responsible for the rising slope and peak amplitude of APs. Inactivation of Na^+^ channels leads to attenuation of Na+ ion driving force into the cell which results in slower rise slope and reduced amplitude of APs during EA. 

Our findings revealed that application of 900 pulses of 1 Hz LFS at the beginning of EA could significantly return RMP to 75% of its baseline value and prevented the AP amplitude decrement. LFS also attenuated changes in the rising and decay slopes of APs generated during EA. These findings might suggest that LFS exerted its inhibitory effects on the hyperexcitability during EA by affecting the kinetics of voltage gated Na^+^ and K^+^ channels.

AP generation can be affected by the synaptic excitation- inhibition ratio in neuronal circuits. An imbalance in the excitation-inhibition ratio is linked to seizure activity (36). Some studies have shown that overstimulation of glutamatergic and attenuation of GABAergic synaptic transmissions can lead to neuronal hyperexcitability in animals (37) and brain slices (38). It has been reported that the decrease in frequency of spontaneous and miniature inhibitory post synaptic currents (IPSCs) in hippocampal CA1 area of epileptic rats led to a three-fold increase in the excitation-inhibition ratio and consequence two-fold increase in AP frequency (37). Therefore, changes in synaptic transmission, which could affect the generation of AP, might be considered as a possible mechanism for the inhibitory action of LFS on EA and changes of AP features during EA.

Administration of LFS at the beginning of EA reduced the EA-induced changes in AP properties. This effect was augmented by increasing the number of LFS pulses from 600 to 900. In addition, phase plot analysis is a suitable method to evaluate neuronal excitability during EA.
